# Role of Episamarcandin in Promoting the Apoptosis of Human Colon Cancer HCT116 Cells through the PI3K-Akt Signaling Pathway

**DOI:** 10.1155/2021/9663738

**Published:** 2021-11-02

**Authors:** Haiying Zhang, Jianan Sun, Ruoting Ma, Shengjun Zhao

**Affiliations:** ^1^Department of Pharmacy, Traditional Chinese Medicine Hospital Affiliated to Xinjiang Medical University, Urumqi 830000, China; ^2^Department of Pharmacy, The Fourth Clinical College of Xinjiang Medical University, Urumqi 830000, China

## Abstract

This study identifies the active ingredients of *Ferula sinkiangensis* and investigates the role and mechanism of episamarcandin in colon cancer cells. The silica gel column chromatography was utilized to separate the chemical components of *Ferula sinkiangensis*. Sephadex LH-20 and semipreparative HPLC were adopted for further separation and purification. The compound episamarcandin showed good anticolon cancer activity among the 13 monomeric compounds obtained. Its effects on the apoptosis, cell cycle, and invasion and migration of colon cancer HCT 116 cells and PI3K-Akt signaling pathway were further investigated. The results showed that, similar to positive control cisplatin, episamarcandin inhibited the proliferation, promoted the apoptosis, arrested cells at G0/G1 phase, and suppressed migration and invasion of HCT 116 cells. A large number of apoptotic HCT 116 cells were observed under a transmission electron microscope. Fluorescence real-time quantitative PCR and western blot analysis showed that episamarcandin increased the expression of PTEN, p53, and Bax and decreased the expression of P-Akt, Akt, mTOR, Bcl-xl, and Bcl-2. Conclusively, episamarcandin may inhibit cell proliferation, migration, and invasion and promote the apoptosis of human colon cancer HCT 116 cells possibly through the PI3K-Akt signaling pathway.

## 1. Introduction

*Ferula* (about 150 species) belongs to *Peucedaneae* of *Apiaceae*, mainly distributed in Central Asia, such as Iran, Pakistan, Turkey, and the former Soviet Union. There are 31 species in China [1]. The Chinese Pharmacopoeia (2015 edition) includes the resin of *Ferula sinkiangensis* or *Ferula fukanensis* [2]. *Ferula sinkiangensis* is mainly distributed in Yining County of Xinjiang and is endemic to China.

Uygur and Kazakh people in Xinjiang, China, have used the root or oleoresin Asafoetida to treat cancers, such as esophageal cancer, colon cancer, and uterine cancer [3]. In the past 20 years, plants of *Ferula* have attracted much attention due to their anti-inflammatory, anticancer, antiangiogenic, anti-P-glycoprotein, and antiviral activities. Studies have reported that plants of *Ferula* can inhibit proliferation and induce apoptosis and cell cycle arrest in human colon cancer HCT 116 cells, human glioma U87 cells, Raji lymphoma cells, cervical adenocarcinoma HeLa cells, and breast cancer MCF-7 cells as well as reducing the metastasis of breast cancer to lungs, livers and kidneys in animal models [4–6]. Our research group has also reported the antitumor and antioxidant activities of *Ferula sinkiangensis* [7–10]. We have demonstrated that the ethyl acetate fraction of *Ferula sinkiangensis* plays important role in inhibitory effect on colon cancer cells in *vitro* and in *vivo*. However, the specific antitumor active ingredients and mechanism of action remain unclear.

The PI3K-Akt signaling pathway plays a central role in promoting cell proliferation, cell movement, invasion, and metastasis, inhibiting cell apoptosis and improving cell hypoxia tolerance [11, 12]. The PI3K-Akt signaling pathway is activated when stimulated by extracellular signals such as cytokines, growth factors, and hormones. Then, the downstream signaling molecule Akt is phosphorylated, which further activates or inhibits its downstream target proteins. PI3K -Akt pathway is usually overactivated in tumor tissues, leading to abnormal proliferation of tumor cells and a decrease in the number of apoptosis [13]. PI3K-Akt pathway can also promote the formation of vascular endothelial cells and affect the resistance of tumors to drugs [14, 15].

Herein, we first identified the active ingredients of *Ferula sinkiangensis* by silica gel chromatography, Sephadex LH-20, and semipreparative high performance liquid chromatography (HPLC). Cisplatin, a widely used therapeutic drug for cancer, was used as a positive control in this study. MTT and SRB (sulforhodamine B) methods were used to verify the inhibitory effect of the 13 obtained monomer compounds on the proliferation of human colon cancer HCT 116 cells, which showed that the sesquiterpene coumarin compound episamarcandin had a significant inhibitory effect on HCT 116 cells. Then, we evaluated the role and mechanism of episamarcandin in colon cancer cells. Cell proliferation, migration and migration, apoptosis, and cell cycle of HCT116 cells after treatment with episamarcandin were assessed. The apoptotic cell morphology was observed under a transmission electron microscope. Fluorescence real-time quantitative PCR and western blot were used to detect the expression levels of key genes of PI3K-Akt signaling pathway and apoptosis-related proteins. Our findings support that episamarcandin may serve as a potential alternative drug for the treatment of colon cancer.

## 2. Materials and Methods

### 2.1. Separation and Preparation of Episamarcandin

Normal phase silica gel column chromatography was utilized to separate episamarcandin. First, 460 g extract was extracted from 2 kg of *Ferula sinkiangensis* resin with 95% ethanol. Then, silica gel column was eluted with a petroleum ether-acetone system gradient (petroleum ether: acetone = 10 : 1, 9 : 1, 8 : 2, 7 : 3, and 6 : 4). The collected eluent was detected at 254 nm and 365 nm wavelengths, developed with 10% H_2_SO_4_-ethanol solution with thin layer chromatography, and then combined to obtain 9 fractions. MTT and SRB assays found that seven fractions (Fr. 73∼104, Fr. 105∼198, Fr. 199∼317, Fr. 368∼397, Fr. 398∼433, Fr. 434∼486, and Fr. 487∼529) showed strong inhibitory effect on human colon cancer HCT 116 cells with an inhibition rate over 80% (Table 1). After analysis by semipreparative HPLC and gel chromatography of these active fractions, 13 monomer compounds were obtained, which included fekolone, sinkianone, lehmannolone, fekrynol, colladonin, feselol, compound 7, lehmannolol, episamarcandin, isosamarcandin, sinkiangenorin E, compound 12, and sinkiangenorin F. Episamarcandin had the best effect on HCT 116 cells (Table 2). The ^13^CNMR spectrum and ^1^H-NMR spectrum were utilized for chemical structure analysis of active compound episamarcandin.

### 2.2. Cell Line

Human colon cancer HCT 116 cells were purchased from the Institute of Cell Research, Chinese Academy of Sciences (Shanghai, China). They were cultured in RPMI l640 medium (HyClone).

### 2.3. MTT Assay

HCT 116 cells were seeded into a 96-well plate at the concentration of 2 × 10^5^ cells•mL^−1^. After culturing for 24 h, 200 *μ*L episamarcandin was added to each well at the concentration of 300 *μ*g•mL^−1^, 150 *μ*g•mL^−1^, 75 *μ*g•mL^−1^, 37.5 *μ*g•mL^−1^, 18.75 *μ*g•mL^−1^, and 9.375 *μ*g•mL^−1^. The cells were cultured for another 24 h, and then MTT (100 *μ*L) was added to each well. After incubation for 3 to 4 h, 150 *μ*L DMSO was added followed by shaking for 15 mins. The optical density (OD) at 490 nm was measured with microplate reader (Multiskan Spectrum, Thermo Scientific, USA). The proliferation inhibition rate was calculated by the formula of inhibition rate (%) =  (OD (control group)–OD (episamarcandin group))/ (OD (control group)-OD (blank group)) × 100%. According to the proliferation inhibition rate of each concentration, the IC_50_ was calculated by IC_50_ calculation model in GraphPad Prism 5.

### 2.4. SRB Assay

Cells were treated as described in MTT assay. After treatment, 0.4% SRB was added and incubated for 30 min. After rinsing four times with 1% acetic acid, 150 *μ*L Tris-base was added and incubated for 10 min on a shaker. The OD at 510 nm was determined using a microplate reader (Multiskan Spectrum, Thermo Scientific, USA). Finally, calculation of cell inhibition rate (inhibition rate (%) =  (OD (control group)–OD (episamarcandin group))/ (OD (control group)-OD (blank group)) × 100%) and IC_50_ was performed.

### 2.5. Wound Healing Assay

HCT 116 cells were plated into a 6-well plate at the concentration of 1 × 10^5^ cells•mL^−1^. An autolaved 10 *μ*L sterile pipette tip was used to scratch lightly on the bottom of the culture plate three times per well. The scratch distance (width between scratch lines) at 0 h was recorded under an inverted microscope (IX-71, Olympus, Tokyo, Japan). Episamarcandin (50 *μ*g•mL^−1^) and the positive control cisplatin (30 *μ*g•mL^−1^) (Jiangsu Hansoh Pharmaceutical Group Co., Ltd., China) was added to each well. At 24 h and 48 h of incubation, the scratch distance (width between scratch lines) was measured, respectively.

### 2.6. Flow Cytometry Analysis

HCT 116 cells were seeded in a 6-well plate at the concentration of 1 × 10^5^ cells•mL^−1^ and cultured for 24 h. For cell apoptosis analysis, episamarcandin (50 *μ*g•mL^−1^, 25 *μ*g•mL^−1^and 12.5 *μ*g•mL^−1^) and cisplatin (30 *μ*g•mL^−1^) were added and incubated for 24 h. The cells were collected, resuspended, and incubated with Annexin V-FITC and PI (abs50001-100T, Absin, Shanghai, China). Cell apoptosis was analyzed on the flow cytometer (Guavaeasycyte 8HT, Millipore, USA). The total apoptosis rate was defined as the sum of the early apoptosis rate and late apoptosis rate.

For cell cycle analysis, cells were cultured with episamarcandin (50 *μ*g•mL^−1^) and cisplatin (30 *μ*g•mL^−1^) for 24 h. The cells were collected and fixed with 75% ethanol at 4°C overnight. Then, cells were incubated with RNase and PI for 30 min at 4°C in the dark. The flow cytometer (Guava easyCyte 8HT, Millipore, USA) was used to analyze cell cycle.

### 2.7. Morphology Observation with Transmission Electron Microscope

HCT 116 cells were seeded in a 6-well plate at the concentration of 1 × 10^5^ cells•mL^−1^. After culture for 24 h, episamarcandin (50 *μ*g•mL^−1^) and cisplatin (30 *μ*g•mL^−1^) were added. After incubation for 24 h, the cells were collected and fixed with 2.5% glutaraldehyde. After rinsing, the sample was dehydrated with acetone, treated with 1% osmium acid at room temperature for 2 h, embedded in epoxy resin, cut into ultrathin sections, and stained with lead-uranium. Finally, the sample was observed under an electron microscope (Tecnai G2 Spirit 120 kV, Thermo Scientific, USA).

### 2.8. Transwell Assay

HCT 116 cells were seeded in a 6-well plate at the concentration of 1 × 10^5^ cells•mL^−1^. Episamarcandin (12.5, 25, and 50 *μ*g•mL^−1^) and cisplatin (30 *μ*g•mL^−1^) were added and incubated for 48 h. Then, cells were collected, resuspended in serum-free medium, and seeded into the upper chamber of the Transwell plate (200 *μ*L each well). For invasion analysis, the upper chamber was precoated with Matrigel. The 200 *μ*L medium containing 10% fetal bovine serum was added to the lower chamber. After another 48 h incubation, 4% paraformaldehyde was added to lower chamber to fix the cells. After that, cells were stained with crystal violet for 30 min. The number of migrated and invaded cells was recorded.

### 2.9. Fluorescence Real-Time Quantitative PCR

HCT 116 cells were treated with episamarcandin (12.5, 25, and 50 *μ*g•mL^−1^) for 24 h. Then, cells were collected, and total RNAs were extracted from cells with TRIzol® Reagent (Invitrogen). The cDNA was obtained by reverse transcription with FastQuant RT Kit (TIANGEN, China). SYBR Select Master Mix (ABI, 4472920) was used for fluorescence real-time quantitative PCR. The primer sequences for *EGFR* (epidermal growth factor receptor)*, PTEN* (phosphatase and tensin homolog deleted on chromosome ten)*, PI3Kp85, Akt1, mTOR* (mammalian target of rapamycin)*, Bcl-xl, p53, Bcl-2, Bax, PI3Kp110δ*, and *β-actin* are shown in Table 3. Fluorescence real-time quantitative PCR was performed on ABI7500 system (7500 Fast Real-Time PCR System, ABI, USA). Prepared cDNA sample was placed in a water bath at 50°C for 2 min, and in another water bath at 95°C for 2 min. After predenaturation, the cDNA sample was moved in a water bath at 95°C for 15 sec and in another water bath at 60°C for 60 sec. Then, the sample was transferred to the PCR amplification machine for 30 cycles in total. The 2^−△△Ct^ method was used to calculate the level of each gene. *β*-Actin was used as internal reference.

### 2.10. Western Blot

After treatment with episamarcandin (12.5, 25, and 50 *μ*g•mL^−1^) for 24 h, cells were collected. Total proteins were extracted after lysis with RIPA. After SDS-PAGE electrophoresis, the protein samples were transferred to membrane. The membrane was probed with PTEN Antibody (CST, #9552S), p53 Antibody (CST, #9282S), Bcl-xl Antibody (CST, #2762S), mTOR Antibody (CST, #2972S), AKT Antibody (CST, #9272S), P-AKT Antibody (CST, #4060S), Bcl-2 Antibody (CST, #2876S), Bax Antibody (CST, #2772S), and *β*-Actin (Abcam, ab8226). The secondary antibody included #31430, Pierce Goat Anti-Mouse IgG (*H* + *L*), Peroxidase Conjugated, Thermo Scientific; #31460, Pierce Goat Anti-Rabbit IgG (*H* + *L*), Peroxidase Conjugated, Thermo Scientific. After color development, the grey value of each band was measured with ChemiScope mini chemiluminescence system (Chemiscope 3000, Clinx Science Instruments Co., Ltd, Shanghai, China).

### 2.11. Statistical Methods

SPSS17.0 statistical software was used with all data expressed as mean ± standard deviation. One-way ANOVA was used for multiple comparisons followed by LSD method for pairwise comparison. Independent sample *t*-test was used to compare the differences between two groups. *P* < 0.05 indicates that the difference is statistically significant.

## 3. Results

### 3.1. The ^13^CNMR Spectrum and ^1^H-NMR Spectrum Parameters of Episamarcandin

**Figure 1:**
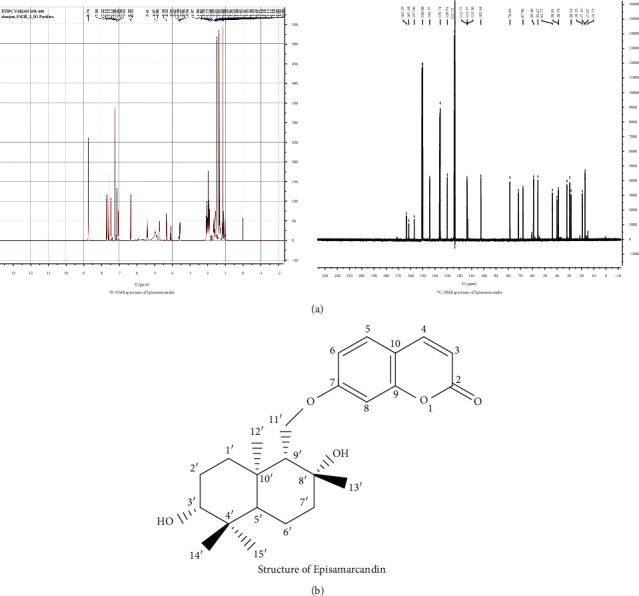
Structure of episamarcandin. (a) ^1^H-NMR and ^13^CNMR spectrum of episamarcandin. (b) Episamarcandin structure.

The chemical structure of episamarcandin was analyzed by ^13^CNMR spectrum and ^1^H-NMR spectrum. The structure of active compound episamarcandin was identified and shown in Figure 1. The ^1^H-NMR parameters were as follows: ^1^H-NMR (C_5_D_5_N, 400 MHz) *δ*: 1.44 (1H, *m*, H-1′a), 2.08 (1H, *m*, H-1′b), 1.87 (1H, *m*, H-2′a), 1.80 (1H, *m*, H-2′b), 3.56 (1H, dd, *J* = 11.2, 4.0 Hz, H-3′), 1.16 (1H, brd, *J* = 12.0 Hz, H-5′), 1.95 (1H, *m*, H-6′a), 1.74 (1H, *d*, *J* = 12.0 Hz, H-6′b), 1.78 (1H, ov., H-7′a), 2.03 (1H, *m*, H-7′b), 1.59 (1H, *d*, *J* = 4.5 Hz, H-9′), 4.08 (1H, dd, *J* = 14.4, 7.2 Hz, H-11′a), 4.33 (1H, *d*, *J* = 9.6 Hz, H-11′b), 1.49 (3H, *s*, H-12′), 1.37 (3H, *s*, H-13′), 1.16 (3H, *s*, H-14′), 1.32 (3H, *s*, H-15′), 6.34 (1H, *d*, *J* = 9.6 Hz, H-3), 7.69 (1H, *d*, *J* = 9.6 Hz, H-4), 7.45 (1H, *d*, *J* = 8.8 Hz, H-5), 7.05 (1H, *d*, *J* = 8.8 Hz, H-6), and 7.12 (1H, brs, H-8). The ^13^CNMR parameters were as follows: ^13^CNMR (C_5_D_5_N, 100 MHz) *δ*: 161.4 (C-2), 113.8 (C-3), 144.4 (C-4), 129.9 (C-5), 113.6 (C-6), 163.4 (C-7), 102.4 (C-8), 157.0 (C-9), 113.3 (C-10), 38.8 (C-1′), 28.5 (C-2′), 78.6 (C-3′), 40.1 (C-4′), 55.7 (C-5′), 19.3 (C-6′), 43.8 (C-7′), 71.6 (C-8′), 59.3 (C-9′), 38.7 (C-10′), 68.0 (C-11′), 32.0 (C-12′), 29.6 (C-13′), 17.1 (C-14′), and 17.2 (C-15′) (Table 4).

### 3.2. Episamarcandin Inhibits the Proliferation of the Human Colon Cancer HCT116 Cells

**Figure 2:**
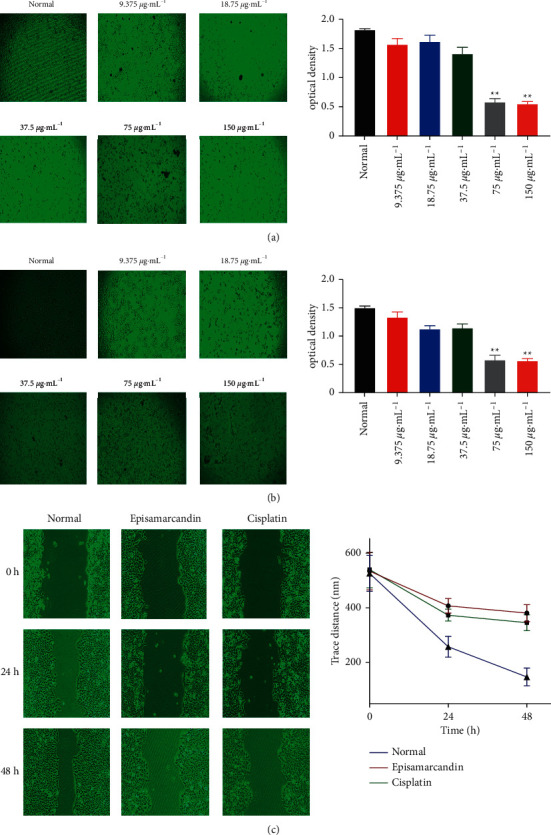
The inhibition of proliferation of human colon cancer cell HCT 116 by episamarcandin. Episamarcandin (9.375 *μ*g•mL^−1^, 18.75 *μ*g•mL^−1^, 37.5 *μ*g•mL^−1^, 75 *μ*g•mL^−1^, 150 *μ*g•mL^−1^, and 300 *μ*g•mL^−1^) was used to treat HCT 116 cells. (a) The proliferation of HCT 116 was detected with MTT method. The cell morphology was shown on the left panel and the statistical results were shown in the right panel. (b) The proliferation of HCT 116 was detected with SRB assay. The cell morphology was shown on the left panel and the statistical results were shown in the right panel. (c) Wound healing assay for the effect of episamarcandin on the migration of HCT 116 cells. Compared with normal control, ^*∗∗*^*P* < 0.01.

The MTT and SRB methods were used to determine the effect of episamarcandin on proliferation of HCT116 cells. The IC_50_ of episamarcandin was determined as 26.06 *μ*g•mL^−1^ and 29.71 *μ*g•mL^−1^, respectively. After 24 h of episamarcandin intervention, the cell shapes gradually changed from spindle shapes to oval ones (Figures 2(a) and 2(b)). Some of them were detached and became suspended cells. Statistically, the inhibition rate of HCT 116 cells was significantly increased with increased drug concentration, suggesting that the proliferation rate was significantly reduced (Figures 2(a) and 2(b)). This result implies that episamarcandin could inhibit proliferation of human colon cancer cells.

### 3.3. Episamarcandin Inhibits Migration of the Human Colon Cancer HCT116 Cells

Cell migration was assessed with wound healing assay. After 24 h and 48 h of episamarcandin incubation, the width between scratch lines of the control group was shorter than that of episamarcandin and cisplatin groups (Figure 2(b)), suggesting that the wound healing degree of episamarcandin and cisplatin group was lower. The 24 h scratch distance of episamarcandin group and normal group was 407.3 ± 5.4 nm and 257.06 ± 7.6 nm, and the 48 h scratch distance was 380.3 ± 6.3 nm and 147.0 ± 6.5 nm, respectively. The migration rate of the episamarcandin group slowed down significantly compared with the control group (*P* < 0.05). The results suggest that episamarcandin has an inhibitory effect on the migration of HCT 116 cells.

### 3.4. Episamarcandin Promotes Apoptosis of the Human Colon Cancer HCT116 Cells

**Figure 3:**
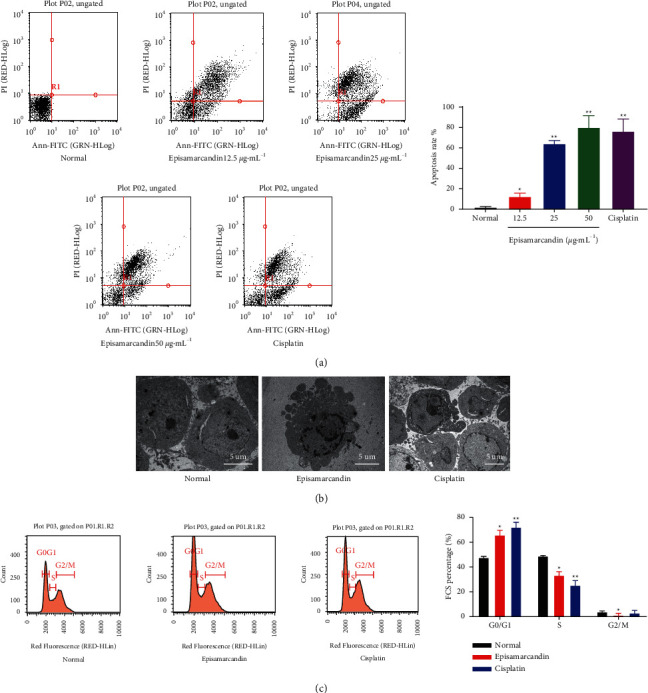
Episamarcandin induces apoptosis and cell cycle arrest of human colon cancer HCT 116 cells. (a) Flow cytometry was used to detect apoptosis. Representative and quantitative results were shown. (b) Cell morphology observed under transmission electron microscope. Magnification: 400x. (c) Flow cytometry was used to analyze cell cycle. Representative and quantitative results were shown. Compared with normal control, ^*∗*^*P* < 0.05,  ^*∗∗*^*P* < 0.01.

After 24 h of episamarcandin intervention, total apoptotic rate of human colon cancer HCT116 cells was analyzed by flow cytometry. Compared with control group, the total apoptotic rate of the group treated with episamarcandin at the concentration of 12.5 *μ*g•mL^−1^, 25 *μ*g•mL^−1^, and 50 *μ*g•mL^−1^ was significantly higher (Figure 3(a)) (*P* < 0.01). Thus, episamarcandin could significantly promote the apoptosis of human colon cancer HCT116 cells.

### 3.5. Morphology and Structure Damage of HCT 116 Cells by Transmission Electron Microscope

After treatment with episamarcandin (50 *μ*g•mL^−1^) for 24 h, the cells were observed by transmission electron microscope (Figure 3(b)). The cells in control group were in round shape with many microvilli on the surface, evenly distributed organelles, large nuclei, complete nuclear membrane, and nucleolus. In episamarcandin and cisplatin groups, the cells showed increased cytoplasmic density unevenly distributed around the cell membrane, incomplete cell membrane, invisible nucleoli, and many vacuoles and apoptotic bodies. The results showed that episamarcandin induced obvious apoptosis of human colon cancer HCT 116 cells.

### 3.6. Episamarcandin Arrests Cell Cycle of Human Colon Cancer HCT 116 at G0/G1 Phase

After the intervention of episamarcandin, cell cycle of HCT 116 cells was analyzed with flow cytometry. Compared with control group, the number of cells in G0/G1 phase was significantly increased, and the number of cells in S and G2/M phases was significantly reduced in episamarcandin group (Figure 3(c)). This effect was similar to that of cisplatin group. The results indicate that episamarcandin could arrest the cell cycle of HCT 116 cells at G0/G1 phase.

### 3.7. Effect of Episamarcandin on HCT 116 Cell Migration and Invasion

**Figure 4:**
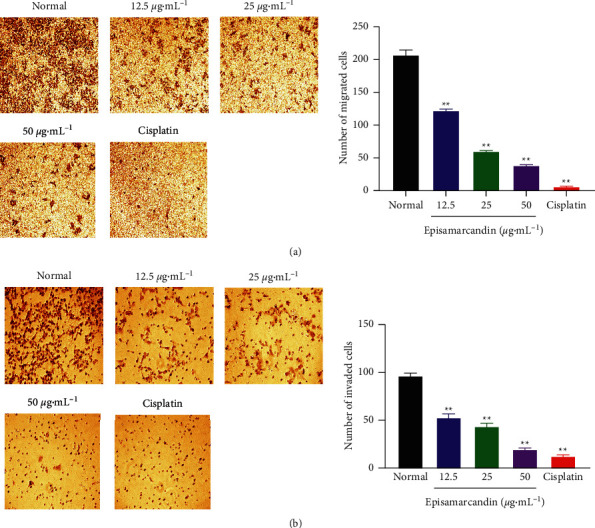
The inhibition of episamarcandin against HCT 116 cell migration and invasion. Transwell assay detected cell migration and invasion after treatment with episamarcandin (12.5 *μ*g•mL^−1^, 25 *μ*g•mL^−1^, and 50 *μ*g•mL^−1^) and cisplatin for 48 h. (a) The number of migrated cells. (b) The number of migrated cells. Compared with normal control, ^*∗∗*^*P* < 0.01.

Transwell assays were applied to monitor the effect of episamarcandin on HCT 116 cell migration and invasion. The results showed that compared with normal control, episamarcandin (12.5 *μ*g•mL^−1^, 25 *μ*g•mL^−1^, and 50 *μ*g•mL^−1^) and cisplatin had significantly lower numbers of migrated (Figure 4(a)) and invaded cells (Figure 4(b)). This indicates that episamarcandin could inhibit the migration and invasion of HCT 116 cells.

### 3.8. Effect of Episamarcandin on Expression of Key Genes of PI3K-Akt Pathway

**Figure 5:**
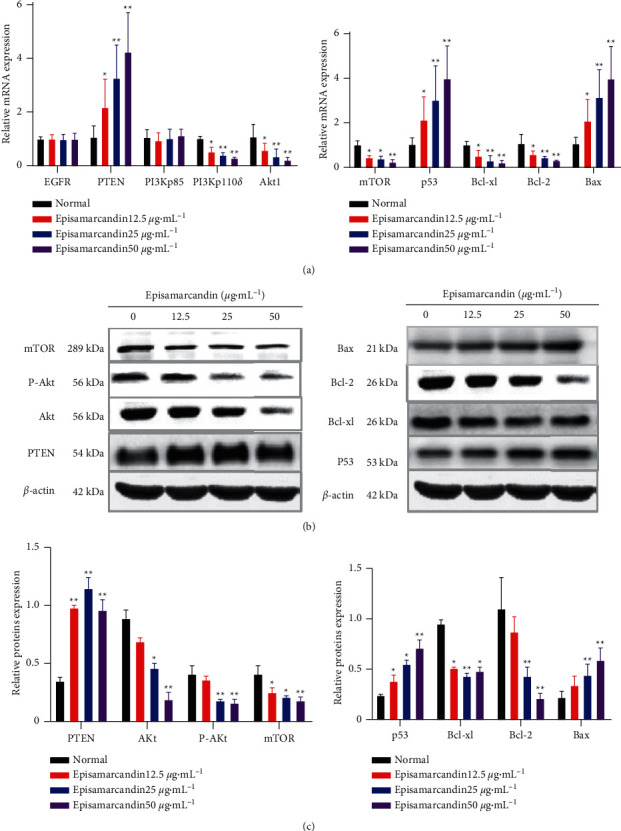
The expression of PI3K-Akt pathway related genes and proteins in HCT 116 cells. Cells were treated with episamarcandin for 24 h. (a) Fluorescence real-time quantitative PCR was performed to detect mRNA expression. (b) Western blot was performed to detect protein expression. Compared with normal control, ^*∗*^*P* < 0.05,  ^*∗∗*^*P* < 0.01.

The fluorescence real-time quantitative PCR was performed to detect the mRNA levels of key genes of PI3K-Akt pathway. Compared with the control group, the gene expression of *EGFR* and *PI3Kp85* in HCT 116 cells after episamarcandin intervention did not change significantly (*P* > 0.05) (Figure 5(a)). However, there was significant increase in expression of of *p53*, *PTEN, PI3Kp11*0*δ*, and *Bax* genes (*P* < 0.05), while significant decrease in gene expression of *Akt1*, *Bcl-xl*, *mTOR*, and *Bcl-2* (*P* < 0.05). In total, episamarcandin shows inhibitory effect on the PI3K/Akt signaling pathway.

### 3.9. Effect of Episamarcandin on Expression of Key Proteins of PI3K-Akt Pathway

Based on the results of gene expression by fluorescence real-time quantitative PCR, the protein levels of *PTEN*, *mTOR*, *Akt1*, *Bcl-xl*, *Bax*, *Bcl-2*, and *p53* with significant changes were further studied by western blot. Compared with the normal control group, the protein expressions of PTEN, p53, and Bax were significantly increased (*P* < 0.05), whereas those of Akt, p-Akt, Bcl-xl, mTOR, and Bcl-2 were significantly reduced (*P* < 0.05) in episamarcandin group (Figure 5(b)). The trend was consistent with that of fluorescent real-time quantitative PCR. This data further verified the inhibitory effect of episamarcandin on the PI3K/Akt signaling pathway.

## 4. Discussion

The cell-level drug screening model has the advantages of less material consumption, clear drug action mechanism, and large screening scale. In this study, the MTT method, SRB assay, scratch healing test, and Transwell assay were used to study the antitumor effect of the compound episamarcandin in *vitro*, which showed that the compound episamarcandin had inhibitory effect on cell proliferation, migration, and invasion of HCT116 cells, and it could be used as a potential cell growth inhibitor of human colon cancer cells.

Many studies have shown that inducing tumor cell apoptosis plays a major role in tumor treatment [16, 17]. Transmission electron microscope has been adopted as the primary method to observe the morphology of apoptotic cells [18]. It can identify the fine structure smaller than 0.2 nm that cannot be observed under the optical microscope. In this study, under transmission electron microscope, the electron density of the cytoplasm of HCT 116 cells increased after the intervention with the compound episamarcandin and cisplatin with uneven thickness and incomplete cell membrane. The apoptotic bodies were observed. This observation showed that episamarcandin may induce the apoptosis of HCT 116 cells. Consistently, the results of flow cytometry in this study showed that the compound episamarcandin promoted the apoptosis of colon cancer HCT 116 cells.

Tumor cells are featured by immortal proliferation. Therefore, to study cell proliferation and apoptosis, cell cycle has to be monitored. The results of this study showed that compared with control group, the number of HCT 116 cells in G0/G1 phase increased significantly, while that in the S phase and G2/M phase decreased significantly after intervention with episamarcandin and cisplatin. Thus, episamarcandin arrested the cell cycle of HCT 116 cells at G0/G1 phase, thereby further inhibiting the proliferation of HCT 116 cells.

PI3K-Akt-mTOR signaling pathway is one of two downstream signaling pathways of EGFR, which can promote colon cancer cell proliferation, prolong cell survival, inhibit cell apoptosis, and participate in angiogenesis, leading to invasion and metastasis of colon cancer [19]. Our study showed that there was no statistically significant change in the expression of *EGFR* gene between the episamarcandin group and the control group. This suggests that episamarcandin may play a role in colon cancer HCT 116 cells in an EGFR independent way.

The PI3K-Akt signaling pathway plays a vital regulatory role in cell proliferation and apoptosis [20]. It is usually overactivated in tumor tissues, leading to abnormal proliferation of tumor cells and reduced level of apoptosis [21]. PTEN can dephosphorylate PIP3 and inhibit PI3K activation, which negatively regulates the PI3K-Akt signaling pathway. It has been reported that the expression rate of PTEN in colon cancer tissues is significantly lower than that in normal mucosal tissues adjacent to the cancer, indicating that the decrease of PTEN is closely related to the proliferation and invasion of colon cancer cells [22]. Akt is also known as protein kinase B and is the main downstream signaling molecule of PI3K. Activated Akt further acts on its downstream target proteins through phosphorylation and then plays its role in regulating cell proliferation, differentiation, migration, and glucose metabolism [23, 24]. TOR is one of downstream target genes of Akt. It has been reported that abnormal activation of mTOR can cause cell cycle activation, thereby promoting tumor formation and tumor cell invasion, metastasis and formation of blood vessels [25]. Bcl-2 family proteins are important components of the mitochondrial apoptosis pathway and play an important role in regulating the function of mitochondria and the release of cytochrome C. The Bcl-2 family proteins mainly include three categories: antiapoptotic protein subfamily (Bcl-2, Bcl-xl, and Mcl-1); proapoptotic protein subfamily (Bax, Bak, and Bok); other proapoptotic proteins (Bid, Bim, and Puma). Bcl-xl has an independent effect on inhibiting cell apoptosis and can directly prevent apoptosis by interfering with the activity of caspase-3 [26]. Bax protein can combine with Bcl-2 to form dimers and coordinately regulate cell apoptosis. If the ratio of Bax/Bcl-2 is increased, Bax-Bax homodimers will increase, which in turn induces cell apoptosis [27]. The p53 gene is also a vital tumor suppressor gene. When the chromosomal DNA of a cell is damaged in the G1 phase, the transcription activity of p53 is enhanced to prevent further cell proliferation [28]. In addition, p53 can also enhance the arrest of tumor cell cycle by regulating other genes and proteins [29]. Our study showed that compared with the normal control group, the episamarcandin and cisplatin groups had significantly upregulated expression levels of PTEN, p53, and Bax, whereas they significantly downregulated expression levels Akt, Bcl-xl, mTOR, and Bcl-2.

It is reported that natural compounds such as calebin A and resveratrol can induce colon cancer cell apoptosis and prevent colorectal cancer metastasis by targeting SIRT1 and inhibiting NF-*κ*B signaling [30–32]. Resveratrol can enhance the sensitivity of chemotherapeutics and improve the antitumor effect of FU on colorectal cancer cells by upregulating the cell–cell junctions, epithelial-mesenchymal transition, and apoptosis, inhibiting tumor necrosis factor-*β* signaling pathway and downregulating NF-*κ*B [33, 34]. Curcumin can enhance the 5-FU-induced reduction in the proliferation and invasion of colon cancer cells, which may help treat colorectal cancer and overcome drug resistance [35]. It is suggested that natural compounds may have chemotherapy sensitization potential and anticancer properties, and their combined use with chemotherapeutics can enhance their antitumor effects. This study showed that episamarcandin had a good inhibitory effect on colon cancer, but whether it can be synergistically used in combination with cisplatin needs further study.

This study presents some limitations. For example, no in vitro or in situ experiments were performed. Thus, further studies are warranted for verification.

## 5. Conclusions

Our study demonstrates that episamarcandin could arrest cell cycle at the G0/G1 phase, inhibit proliferation, migration, and invasion, and promote the cell apoptosis of colon cancer HCT 116 cells. The underlying mechanism of episamarcandin may be upregulation of PTEN, p53, and Bax, reduction of Akt phosphorylation, and downregulation of mTOR, Bcl-xl, and Bcl-2, which may further lead to the inhibition of HCT 116 cell growth and proliferation and the promotion of apoptosis. Together, episamarcandin from *Ferula sinkiangensis* shows potentially promising inhibition effects against colon cancer HCT 116 cells and may be suitable as combination therapy or supportive therapy for colon cancer.

## Figures and Tables

**Table 1:** Cytotoxicity of compounds against HCT116 and SGC-7901 cell lines.

Compounds	HCT116 IC_50_ (*μ*g·mL^−1^)		SGC-7901 IC_50_ (*μ*g mL^−1^)	
	MTT assay	SRB assay	MTT assay	SRB assay
Fekolone	>50	>50	>50	>50
Sinkianone	>50	>50	>50	>50
Lehmannolone	>50	>50	>50	>50
Fekrynol	31.56 ± 2.68	35.84 ± 2.79	40.41 ± 3.12	45.24 ± 3.76
Colladonin	>50	>50	>50	>50
Feselol	>50	>50	>50	>50
Compound 7	>50	>50	>50	>50
Lehmannolol	>50	>50	>50	>50
Episamarcandin	26.06 ± 1.28	29.71 ± 2.12	30.14 ± 2.76	34.97 ± 3.12
Isosamarcandin	>50	>50	>50	>50
Sinkiangenorin E	>50	>50	>50	>50
Compound 12	40.24 ± 3.94	42.78 ± 4.12	>50	>50
Sinkiangenorin F	>50	>50	>50	>50

**Table 2:** Cytotoxicity of episamarcandin on HCT 116 cells.

Episamarcandin concentration (*μ*g·mL^−1^)	Inhibition rate on HCT 116 cells (%)	
	MTT assay	SRB assay
300	99.46	98.32
150	98.01	95.19
75	97.60	93.39
37.50	37.91	36.17
18.75	19.79	37.91
9.38	18.28	17.08
IC_50_ (*μ*g mL^−1^)	26.06 ± 1.28	29.71 ± 2.12

**Table 3:** Primer sequence for fluorescence real-time quantification PCR.

Gene ID	Primer	Sequence (5′to3′)	Length (bp)
NM_001101	*β*-Actin_F	ATGATGATATCGCCGCGCTC	211
	*β*-Actin_R	TCGATGGGGTACTTCAGGG	

NM_201284	EGFR-F	AGGCACGAGTAACAAGCTCAC	177
	EGFR-R	ATGAGGACATAACCAGCCACC	

NM_000314	PTEN-F	TTTGAAGACCATAACCCACCAC	134
	PTEN-R	ATTACACCAGTTCGTCCCTTTC	

NM_001242466	PI3Kp85-F	ACCACTACCGGAATGAATCTCT	207
	PI3Kp85-R	GGGATGTGCGGGTATATTCTTC	

NM_005163	Akt1-F	GTCATCGAACGCACCTTCCAT	218
	Akt1-R	AGCTTCAGGTACTCAAACTCGT	

NM_004958	mTOR-F	GCAGATTTGCCAACTATCTTCGG	114
	mTOR-R	CAGCGGTAAAAGTGTCCCCTG	

NM_001322240	Bcl-xl-F	GACTGAATCGGAGATGGAGACC	179
	Bcl-xl-R	GCAGTTCAAACTCGTCGCCT	

NM_001126118	p53-F	CAGCACATGACGGAGGTTGT	125
	p53-R	TCATCCAAATACTCCACACGC	

NM_000633.3	Bcl-2-F	GGATAACGGAGGCTGGGATG	103
	Bcl-2-R	GGCCAAACTGAGCAGAGTCT	

NM_001291428.2	Bax-F	CATGGGCTGGACATTGGACT	137
	Bax-R	AAAGTAGGAGAGGAGGCCGT	

NM_001350234.2	PI3Kp110*δ*-F	TGGACTGCCCCATGGAATTC	203
	PI3Kp110*δ*-R	GCAGGTGAACACATAGGCCT	

**Table 4:** The ^1^H-NMR and^13^CNMR parameters of episamarcandin.

	H^1^NMR	^13^CNMR
1	1.44 (1H, *m*, H-1′a)	161.4 (C-2)
2	2.08 (1H, *m*, H-1′b)	113.8 (C-3)
3	1.87 (1H, *m*, H-2′a)	144.4 (C-4)
4	1.80 (1H, *m*, H-2′b)	129.9 (C-5)
5	3.56 (1H, dd, *J* = 11.2, 4.0 Hz, H-3′)	113.6 (C-6)
6	1.16 (1H, brd, *J* = 12.0 Hz, H-5′)	163.4 (C-7)
7	1.95 (1H, *m*, H-6′a)	102.4 (C-8)
8	1.74 (1H, *d*, *J* = 12.0 Hz, H-6′b)	157.0 (C-9)
9	1.78 (1H, ov., H-7′a)	113.3 (C-10)
10	2.03 (1H, *m*, H-7′b)	38.8 (C-1′)
11	1.59 (1H, *d*, *J* = 4.5 Hz, H-9′)	28.5 (C-2′)
12	4.08 (1H, dd, *J* = 14.4, 7.2 Hz, H-11′a)	78.6 (C-3′)
13	4.33 (1H, *d*, *J* = 9.6 Hz, H-11′b)	40.1 (C-4′)
14	1.49 (3H, *s*, H-12′)	55.7 (C-5′)
15	1.37 (3H, *s*, H-13′)	19.3 (C-6′)
16	1.16 (3H, *s*, H-14′)	43.8 (C-7′)
17	1.32 (3H, *s*, H-15′)	71.6 (C-8′)
18	6.34 (1H, *d*, *J* = 9.6 hz, H-3)	59.3 (C-9′)
19	7.69 (1H, *d*, *J* = 9.6 hz, H-4)	38.7 (C-10′)
20	7.45 (1H, *d*, *J* = 8.8 hz, H-5)	68.0 (C-11′)
21	7.05 (1H, *d*, *J* = 8.8 hz, H-6)	32.0 (C-12′)
22	7.12 (1H, brs, H-8)	29.6 (C-13′)
23		17.1 (C-14′)
24		17.2 (C-15′)

## Data Availability

The data used to support the findings of this study are available from the corresponding author upon request.
